# Turning Susceptibility into Strength: A New Era of Durable Resistance in Plants Through Genome Editing

**DOI:** 10.3390/plants14193080

**Published:** 2025-10-05

**Authors:** Shallu Thakur, Simranjot Kaur, Sudeep Adhikari, Prerna Sabharwal, Yuqing Fu, Geoffrey Meru

**Affiliations:** Tropical Research and Education Centre, Horticultural Sciences Department, University of Florida, IFAS, Homestead, FL 33031, USA

**Keywords:** susceptibility genes, CRISPR/Cas9, disease resistance, pathogens, crops, genome editing

## Abstract

In plants, resistance genes (*R*) are key players in combatting diseases caused by various phytopathogens. Typically, resistance relies on detecting a single pathogen-derived molecular pattern. However, *R*-gene-mediated resistance is often race specific, follows the gene-for-gene hypothesis, and can be overcome in field conditions as pathogens evolve. On the contrary, altering plant susceptibility genes (*S*-genes) facilitates compatibility and results in broad and durable resistance. *S*-genes are negative regulators present in plants and exploited by pathogens to facilitate their growth and cause infection. Several studies across crop species have reported manipulation of *S*-genes using genome editing to confer broad spectrum resistance. This review focuses on the plant defense mechanism against biotic stress, *R*-genes vs. *S*-genes, different types/classes of *S*-genes, different tools for *S*-gene discovery, and the use of gene editing technologies to target *S*-genes in addition to their applications, challenges, and future perspectives.

## 1. Introduction

Crop yields are reduced by 40% each year due to biotic stressors such as insect pests and pathogens [1]. Estimates indicate a need to increase food productivity by 70% to feed an exponentially growing global population of 10 billion by 2050 [2]. Achieving this ambitious goal will require not only boosting agricultural production but also minimizing the losses caused by various biotic stresses [1]. With rising global temperatures, the frequency of disease outbreaks is expected to increase, which could hamper productivity and pose major challenges to food security [3]. Therefore, there is a pressing need to explore ways to enhance plants’ tolerance to various biotic stresses.

Biotic stresses in plants are caused by living organisms such as bacteria, viruses, fungi, nematodes, insects, arachnids, and weeds [4]. Plants have naturally evolved to defend themselves against invading pathogens by developing efficient defense mechanisms [5,6,7]. One of the most effective strategies to manage phytopathogens is the development and use of disease-resistant crop varieties. Developing resistant plant varieties usually involves introducing naturally occurring dominant resistance (*R*) genes into crop cultivars. However, resistance sources are available in the wild germplasm, limiting their applicability [8,9]. Furthermore, *R*-gene-mediated resistance is frequently overcome by the emergence of new races that evade recognition by altering their cognate effector proteins [10].

Inactivating susceptibility (*S*) genes, which facilitate pathogen infection and compatibility, offers a promising strategy for durable plant disease resistance [11,12]. Unlike *R*-gene-mediated resistance, *S*-gene inactivation provides broad-spectrum and more reliable resistance. In the year 2002, the concept of an *S*-gene was first explored after the identification of pmr6 (PM resistance) in Arabidopsis. PMR6 was described as “… a novel form of disease resistance based on the loss of a gene required during a compatible interaction …” [13], after which the term “susceptibility gene” was proposed [14]. Inactivation, mutation, or loss of *S*-gene function leads to disease-resistant plants by disrupting compatible plant–pathogen interactions and slowing pathogen growth and disease progression [11,15]. Many studies have demonstrated the exploitation of *S*-genes in conferring disease resistance against various biotic stresses [16]. Among *S*-genes, Mildew Resistance Locus O (*MLO*) is the most extensively studied, with its role in plant immunity conserved across species. To date, approximately 200 *MLO* genes have been identified, grouped into seven conserved clades, with Clade IV (monocots) and Clade V (dicots) most associated with powdery mildew (PM) susceptibility. In barley, loss of function *mlo* alleles have shown durable broad-spectrum resistance against PM for more than five decades, with few pleiotropic effects [17,18]. In addition, similar gene orthologs have been mutated in several crops to confer resistance to PM, including Arabidopsis, wheat, tomato, pea, pepper, grapevine, and strawberry [19,20,21,22,23,24]. This has led to the discovery and characterization of many other *MLO* orthologs in several plant species, such as Arabidopsis (*AtMLO2*, *AtMLO6*, and AtMLO12), barley (*TaMlo-A1*, *TaMlo-B1*, and *TaMlo-D1*), cucumber (*CsaMLO8*), melon (*CmMLO3*, *CmMLO5*, and *CmMLO12*), *C. moschata* (*CmaMLO1*, *CmoMLO2*, *CmoMLO3*, *CmoMLO4*, and *CmoMLO5*), and *C. maxima* (*CmaMLO1*, *CmaMLO2*, *CmaMLO3*, *CmaMLO4*, and *CmaMLO5*), in addition to tomato (*SlMLO1*) and pea (*Er1/PsMLO1*) [19,25,26,27,28].

Another well-characterized *S*-gene is the eukaryotic initiation factor 4E (*eIF4E*), which plays a crucial role in viral RNA replication [29]. Inactivation of this gene (*eIF4E*) leads to resistance against potyvirus, and loss-of-function *eIF4E* mutants confer resistance in tomato, rice, barley, lettuce, melon, pea, and pepper [30]. Mutating either *eIF4E* or *eIF(iso)4E* is often enough to provide potyvirus resistance with minimal or no pleiotropic effects [31]. The *eIF4E* and *MLO* examples highlight that the dependency of pathogens on host factors for infection or replication runs the risk of reaching a dead end in the evolutionary arms race. To overcome this, they must either revert and regain a previously lost function or shift to a different host altogether. This concept highlights the potential of *S*-genes as important targets for resistance breeding.

Previously, RNA interference (RNA-i) and Transcription Activator-Like Effector Nuclease (TALEN)-induced mutagenesis were used to inactivate *S*-genes [22,23,32,33,34]. With advancements in genome editing (GE) technology, clustered regularly interspaced short palindromic repeats (CRISPR)-associated nucleases (Cas9) have been used successfully to modify *S*-genes to obtain effective disease-resistant crops. Owing to its specificity, adaptability, ease of use, and cost and labor effectiveness, this transgene-free technology has the potential to create resistant plants against a plethora of microbes by identifying and targeting *S*-genes in various crop species to tackle the challenge of food security and environmental sustainability [35,36]. This review summarizes plant defense strategies against biotic stress, *R*-genes vs. *S*-genes, different types/classes of *S*-genes, different tools for *S*-gene discovery, and different *S*-genes targeted using CRISPR/Cas9 technology, along with their applications, challenges, and future perspectives.

## 2. Defense Mechanism in Plants Against Biotic Stress

**Figure 1:**
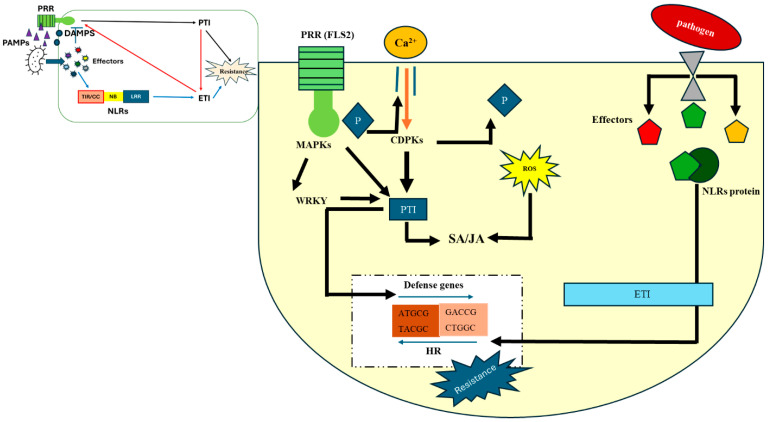
Plant defense mechanisms against biotic stress involve multiple layers of immunity. PAMPs/DAMPs are detected by PRRs on the cell surface, such as FLS2, which trigger PTI through the activation of MAPK cascades or calcium-dependent protein kinases (CDPKs). Effectors produced by bacteria, virus, fungi, and oomycetes that entre the plant cell are recognized by NOD-like receptors (NLRs), initiating effector-triggered immune ETI and leading to HR responses. The letter P indicates phosphorylation, SA/JA—salicylic acid (SA) and jasmonic acid (JA); ROS—Reactive oxygen species.

Plants defend themselves via two tiers of receptors against invading pathogens [37,38]. The first layer consists of transmembrane pattern-recognition receptors (PRRs), which are responsible for detecting conserved pathogen-associated molecular patterns (PAMPs) from microbes and damage-associated molecular patterns (DAMPs) from plants [39]. The first line of defense in plants against pathogens is known as pathogen-triggered immunity (PTI) [40]. The activation of PTI is the essential component of plant innate immunity. The recognition of PAMPs by PRRs triggers a cascade of downstream signaling pathways that activate plant defense responses. As a result, a pathogen’s virulence potential depends on its ability to suppress PTI using effector molecules [41]. Activation of PTI initiates signaling cascades involving mitogen-activated protein kinases (MAPKs), transcriptional reprogramming through transcription factors such as WRKY, and the production of various reactive oxygen species (ROS) in the host plant [42]. These signaling cascades also involve key hormones such as salicylic acid (SA) for biotrophs, and jasmonic acid (JA) and ethylene for necrotrophs to enhance resistance and trigger localized hypersensitive responses (HRs), characterized by programmed cell death at infection sites (Figure 1).

In contrast, effector-triggered immunity (ETI) is activated by specific pathogenic effectors, with their corresponding plant receptors showing high specificity and undergoing strong diversifying selection. Most ETI-associated receptors inside host cells are detected by nucleotide-binding/leucine-rich repeat (NLR) receptors and other cytoplasmic proteins [43,44]. ETI responses are typically stronger and more long lasting than PTI and often lead to HR and programmed cell death [45,46]. In ETI, pathogen effectors, known as avirulence (Avr) proteins, are detected by host NLRs, also referred to as R proteins. In simple terms, Avr proteins function as ligands that specifically bind to corresponding NLRs. Activation of ETI triggers an HR and programmed cell death in infected and surrounding cells, helping to limit pathogen spread. Disease occurs when there is a compatible interaction between plant and pathogen. In many cases, plants achieve durable resistance by modifying genes that are essential for compatibility. These genes, which enable pathogen infection and support compatibility, are known as susceptibility (*S*) genes [11]. Despite these defense mechanisms, a few host susceptibility (*S*) genes follow an inverse gene-for-gene model. The necrotrophic pathogen utilizes effectors to coopt host programmed cell death known as effector-triggered susceptibility (ETS) [37]. The effectors or host-selective toxins (HSTs) directly interact with sensitivity genes, activate plant defense responses including cell death, and lead to susceptibility. ETS is therefore regarded as a pathogen-driven manipulation of host genes and metabolic pathways, representing the opposite of *R*-gene-mediated resistance.

## 3. *R*-Genes vs. *S*-Genes

**Figure 2:**
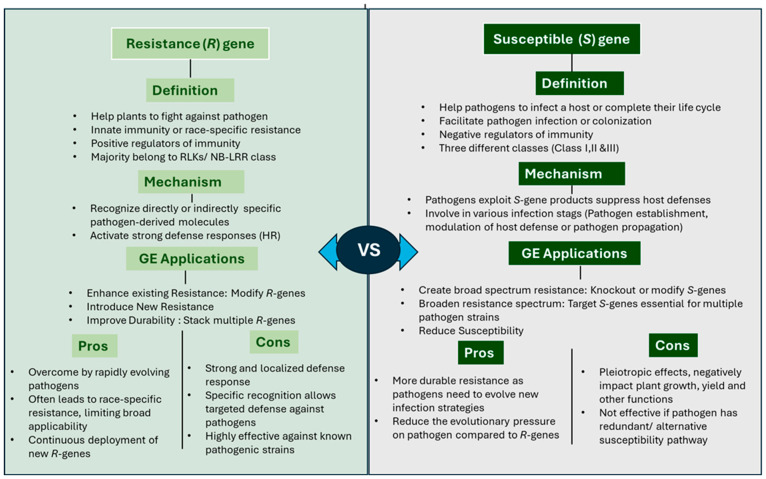
Comparative overview of *R*-genes and *S*-genes, highlighting mechanisms, editing applications, advantages, and limitations.

Plant *R*-genes function by directly or indirectly recognizing conserved pathogen effectors, also known as Avr proteins. Most *R*-genes encode either surface immune receptors such as receptor-like kinases (RLKs) or intracellular immune receptors [47]. Among these, the nucleotide-binding leucine-rich repeat (NB-LRR or NLR) family represents the largest class of *R*-genes, characterized by a nucleotide-binding (NB) domain and a leucine-rich repeat (LRR) domain. Based on their N-terminal structural motifs, NLRs are further categorized into Toll/interleukin-1 receptor (TIR)-NB-LRR and coiled-coil (CC)-NB-LRR subclasses [48]. In traditional breeding, disease resistance in plants has been introduced through the incorporation of *R*-genes. This involves transferring *R*-genes from closely related wild relatives or compatible species into cultivated varieties [49]. Over the years single or multiple *R*-genes have been successfully introduced into different crops [49]. However, this strategy has limitations, as pathogen *Avr* effector proteins frequently mutate, enabling them to evade recognition by host *R*-gene receptors [50,51,52]. Consequently, plant breeders continually search for new *R*-gene sources to combat newly emerging pathogens. In this context, CRISPR/Cas-mediated modification or engineering of known NLRs has emerged as a promising strategy for enhancing disease resistance. Editing regulatory elements within NLRs can improve their activation potential, thereby boosting plant immunity [53]. Additionally, modifying the effector recognition sites of NLRs can confer broad-spectrum disease resistance [53]. For example, targeted mutagenesis of the tomato I2 immune receptor successfully expanded effector recognition and provided broad-spectrum resistance [54,55]. Similarly, precise editing of the LRR domain of NLRs using CRISPR/Cas9 can generate novel resistance traits, as the LRR domain plays a critical role in pathogen recognition specificity [56,57,58] (Figure 2).

*S*-gene editing offers an alternative and effective approach for developing crop cultivars with durable disease resistance. Plant *S*-genes function as negative regulators of immunity and are frequently exploited by pathogens to enhance their proliferation and initiate infection [59]. Since *S*-genes are naturally present in the host plant, introgression of foreign genetic material is not required to develop resistance. Knocking out *S*-genes can confer broad-spectrum disease resistance and prevent pathogens from successfully infecting the host (Figure 2). However, resistance conferred by *S*-gene loss or modification is usually recessive and may come with some fitness costs [12]. In plants, the balance between *R*-genes and *S*-genes largely determines whether a plant is susceptible or resistant to disease. Recent advances in genome editing (GE) technologies have transformed plant science, enabling improvements not only in disease resistance but also in nutritional value and fruit quality traits. Tools such as transcription activator-like effector nucleases (TALENs), zinc-finger nucleases (ZFNs), and especially CRISPR/Cas9 allow precise manipulation of target DNA sequences [60,61,62]. Among these, CRISPR/Cas9 is widely used to study *S*-gene function and develop disease-resistant cultivars. Although recessive inheritance makes *S*-gene-based resistance challenging to incorporate through conventional breeding, GE provides a powerful approach to create durable and broad-spectrum resistance, similar to non-host resistance [63].

## 4. Methods for Identification of *S*-Genes in Plants

**Figure 3:**
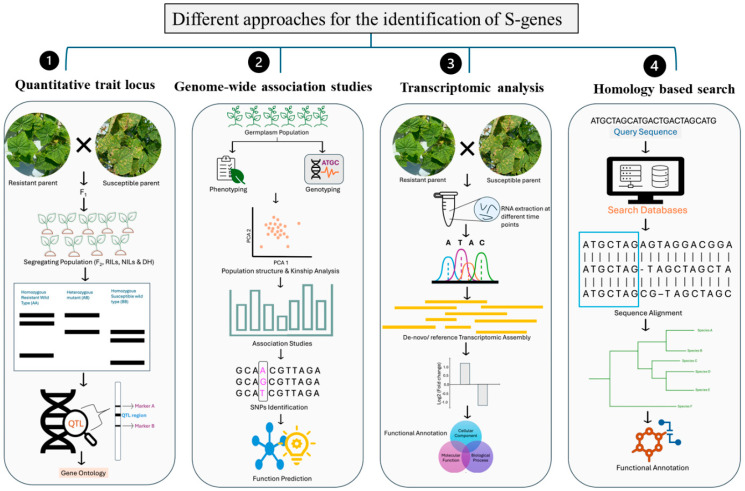
Methods for identifying *S*-genes include QTL mapping, GWAS, transcriptomic analysis, and homology-based searches to discover candidate genes associated with pathogen interactions.

Identifying *S*-genes, deciphering their functions, and mapping their location within the plant genome are essential steps toward engineering broad-spectrum disease resistance and developing improved crop cultivars [64]. As research into plant–pathogen interactions advances, a range of methodologies have been used to identify and characterize these elusive *S*-genes. Here, a detailed description of molecular and computational approaches used for the identification of *S*-genes is presented (Figure 3).

### 4.1. Quantitative Trait Locus (QTL)

QTL mapping is a process of identifying genomic regions linked to variations in quantitative traits, including disease susceptibility [65]. This process involves the development of suitable mapping populations by crossing plants with contrasting phenotypes (e.g., resistant vs. susceptible) followed by genotyping. Finally, by using appropriate statistical packages to perform linkage analysis by using genotyping and phenotypic information, researchers can identify genomic regions responsible for susceptibility [66]. For example, a single major QTL flanked by two AFLP markers (200011_345Dr and 200011_208Br) governing susceptibility to PM in hop (*Humulus lupulus L*) has been identified in co-segregating populations [67]. In *Lathyrus sativus* and *L. cicero*, the *MLO1* gene was recently identified using QTL mapping [68]. Further phylogenetic analysis confirmed that *LsMLO1* and *LciMLO1* belong to Clade V (associated with susceptibility) [68]. Similarly, QTL mapping and RT-PCR revealed *CsGy5G015660* as a strong candidate gene conferring PM resistance in cucumber [66].

### 4.2. Genome-Wide Association Studies (GWASs)

The GWAS approach is a genetic approach used to identify the genetic variants associated with a specific trait across large natural populations [69,70]. GWASs typically identify broad genomic regions linked to susceptibility and fine mapping techniques are then used to narrow these regions and locate the most likely candidate *S*-genes [71]. For instance, a recent GWAS in tea (*Camellia sinensis*) identified the *CsNCED1* gene, which encodes 9-cis-epoxycarotenoid dioxygenase, a key enzyme in abscisic acid (ABA) biosynthesis, as a susceptibility factor for biotic stress. Overexpression of *CsNCED1* led to reduced salicylic acid levels and downregulation of immune-related genes (e.g., *NPR1*, *PR1*, and *WRKY18*), resulting in greater susceptibility to *Spodoptera litura* [72].

### 4.3. Transcriptomic Analysis

Transcriptomics studies are widely used to study gene expression patterns in response to pathogen infection. The transcriptomics profiles of resistant and susceptible genotypes at different time intervals were compared to identify differentially expressed genes, including potential *S*-genes, that play a role in plant defense mechanisms [73,74,75].

For instance, nine *S*-gene homologs of *CAMTA3*, *CNGC4*, *PMR4*, *PMR5*, and *PUX2* (*S*-genes in Arabidopsis have been identified in tobacco using RNA-seq analysis of resistant (BH) and susceptible (XHJ) tobacco cultivars infected with *Phytophthora nicotianae* [76]. Similarly, *CsaMLO1*, *CsaDMR6-2*, and *PMR/DMR* homologs in cucumber have been identified based on transcriptomic data [77].

### 4.4. Homology-Based Search

Initially, an extensive literature review was performed to identify genes implicated in plant–pathogen interactions [78,79,80]. With the increasing availability of extensive genomic resources for a wide range of crop species, identifying homologous or functionally similar genes in the genome of interest has become more feasible. Identifying genes through this approach helps narrow down the pool of potential candidate genes [81]. The BLAST sequence similarity search is a major platform for screening high-level similarity or conserved coding regions. Moreover, various in-silico bioinformatics tools are available with many statistical algorithms or computational methods, for instance, data-mining analysis, hidden Markov analysis, cluster analysis, kernel-based data fusion analysis, machine learning, K-Nearest Neighbors (KNN) classification algorithm, and others. To further investigate their roles in disease resistance, various molecular approaches such as gene expression analysis, knockout (KO) or overexpression studies, and protein–protein interaction assays can be employed. Several tools and databases are now available to facilitate the prediction of protein–protein interactions between candidate *S*-genes and pathogen effectors (Table 1). These platforms primarily incorporate machine learning (ML) and deep learning (DL) models to improve prediction accuracy and efficiency. Using similar approaches, *S*-genes has been identified in arabidopsis, barley, cucumber, tomato, chestnut, cotton, rubber, poplar, apple, grapevines, and many more [19,23,25,26,27,81,82,83]. Together, these approaches offer an effective way to identify and characterize *S*-genes, allowing targeted modifications to strengthen plant resistance and improve disease management strategies.

## 5. Classification and Mechanisms of *S*-Genes

**Figure 4:**
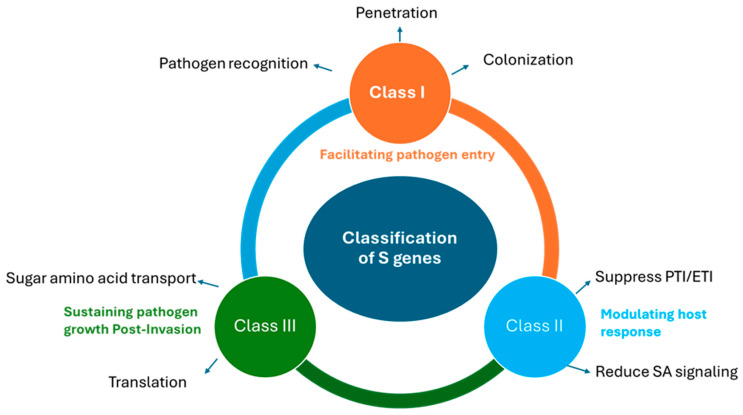
Mode of action of different classes of *S*-genes during pathogen infection.

There are three major classes of *S*-genes based on their mechanism of action (Figure 4).

### 5.1. Facilitating Host Recognition and Entry

*S*-genes enable pathogen recognition, penetration, colonization and initiation of infection through various mechanisms including modifying structural barriers or immune responses. Class I genes are active during the early stages of infection, facilitating pathogen entry and establishing infection through various entry points [11]. For instance, mutants of the *Glossy II* gene in corn have shown reduced susceptibility to PM due to lower levels of very long-chain aldehydes in the leaf cuticles [110]. Similarly, the *ram2* mutant in *Medicago truncatula* has exhibited decreased susceptibility to *Phytophthora palmivora* because of altered cutin composition [111]. In another study, the *irg1* mutant in *Medicago* exhibited reduced epicuticular wax levels on the leaf surface, and the mutants showed decreased susceptibility to fungal pathogens including *Phakopsora pachyrhizi*, *Puccinia emaculata*, and *Colletotrichum trifolii* [112]. Similarly, mutants in Arabidopsis genes *bre1/lacs2*, which are defective in cutin biosynthesis, display increased cuticle permeability. This alteration facilitates the release of antifungal compounds, leading to enhanced resistance against *Botrytis cinerea* [113,114]. The *rat4* mutants in Arabidopsis, with disrupted *CSLA*9 genes encoding a cellulose-like protein, show reduced susceptibility to *Agrobacterium* infection, highlighting the role of cell wall components in pathogen recognition [115,116]. Pathogens also exploit natural openings like stomata for entry, a process regulated by genes such as *LecRK*, *RIN4*, and *AHA1*. Mutants in these genes exhibit reduced bacterial entry [117,118,119]. Membrane genes like *MLO* and *BAX* inhibitor-1 *(BI-1*) are essential for haustoria formation in PM pathogens, and their loss of function confers broad resistance [111,120,121]. Similarly, small GTPases like *HvRACB* in barley and *ROP*6 in Arabidopsis support susceptibility to adapted pathogens while resisting non-adapted ones [122,123]. In rice, genes such as *OsRAC4*, *OsRAC5*, and *OsRACB* are associated with susceptibility to *Magnaporthe oryzae* [124,125]. In *Arabidopsis thaliana*, the ARF-GAP protein AGD5 functions as a susceptibility factor during infection by *Hyaloperonospora arabidopsidis* [126]. These examples collectively highlight the diverse roles of *S*-genes in modulating plant structures and physiological processes to facilitate pathogen infection [11].

### 5.2. Modulating Host Immune Responses

*S*-genes act as negative regulators in plant immunity that suppress host defense signaling, allowing pathogens to evade immune responses. In plant immunity, *S*-genes that play a crucial role in modulating defense responses belong to class 2 genes. These genes, while not directly involved in pathogen resistance, influence the plant’s ability to respond effectively to infections. For instance, in Arabidopsis, the *PMR4* gene encodes callose synthase, which is involved in cell wall reinforcement during pathogen attack. Mutations in *PMR4* lead to enhanced resistance against PM by promoting callose deposition at infection sites, thereby restricting pathogen entry [127]. Similarly, the *WRKY45* transcription factor in rice has been identified as an *S*-gene and the overexpression of *WRKY45-1* increases susceptibility to *Xanthomonas oryzae*, while *WRKY45-2* enhances resistance, highlighting the complex roles of *WRKY* genes in disease susceptibility [128]. In Arabidopsis, the *DMR6* gene functions as an *S*-gene for Downy Mildew (DM). It promotes pathogen growth by degrading SA, a crucial signaling molecule that plays an essential role in enhancing plant defense mechanisms [129,130]. Likewise, begomovirus infection triggers the production of a calmodulin-like protein called rgs-CaM, which acts as a negative regulator of gene silencing. The disruption of RNA silencing machinery by rgs-CaM reduces the transcription of RNA-dependent RNA polymerase 6 (*RDR6*) and promotes the degradation of suppressor of gene silencing 3 (*SGS3*) [131,132]. *RDR6* plays a key role in amplifying RNA silencing signals, while *SGS3* helps maintain their stability and distribution. *RDR6* suppression and *SGS3* degradation by rgs-CaM weaken the RNA silencing pathway, resulting in reduced signal amplification, increased viral RNA accumulation, and more severe infections [131]. These examples highlight the functions of class 2 *S*-genes in plant defense, where their modulation can tilt the balance between susceptibility and resistance.

### 5.3. Sustaining Pathogen Growth Post-Invasion

In plant–pathogen interactions, *S*-genes that facilitate pathogen establishment by providing essential resources belong to class 3 genes. These genes are often recessive and are exploited by pathogens to enhance their virulence through specialized effectors. These effectors modify plant cellular processes to create a more favorable environment for pathogen growth. Plant–pathogen interactions often hinge on the exploitation of host *S*-genes, which pathogens hijack to facilitate their invasion and proliferation. For example, in rice, the *SWEET* family of sugar transporters, such as *SWEET11* and *SWEET13*, are targeted by *Xanthomonas oryzae* to export sugars into the apoplast, providing essential nutrients for the pathogen’s growth [124]. Similarly, in Arabidopsis, the *DMR1* gene, encoding homoserine kinase, is exploited by *Hyaloperonospora arabidopsidis*, which relies on host-derived amino acids for its development [133,134]. In maize, the lipoxygenase gene *ZmLOX3* is involved in the production of oxylipins that promote fungal virulence, however, mutations in *ZmLOX3* can increase resistance to certain pathogens [135]. Barley alcohol dehydrogenase (*ADH*) gene expression is triggered by PM infection, promoting pathogen growth by enhancing anaerobic glycolytic metabolism. In contrast, plants with silenced *ADH* genes show reduced susceptibility to PM. [136]. PM susceptibility genes like *PMR5* and *PMR6* encode for pectate lyases, and mutations in these alleles result in accumulation of pectin in cell walls and reduced pathogen proliferation at later infection stages [13,137]. Plant viruses often hijack host translation machinery for replication; for instance, *eIF4E* and *eIF(iso)4E* play a key role in potyvirus infection by interacting with the viral protein (VPg). Mutations in these factors can confer resistance to potyviruses, but viruses may adapt by acquiring mutations in VPg to interact with alternative *eIF4E* isoforms. In Arabidopsis, a mutated *eIF4* gene restricted the viral movement within the host and resulted in reduced susceptibility to cucumber mosaic virus (CMV) and turnip crinkle virus (TCV) [138]. Additionally, tonoplast membrane proteins TOM1, TOM2, and TOM3 in Arabidopsis are required for efficient multiplication of tobamoviruses, indicating their essential role in the viral life cycle [139,140,141]. The nine necrotrophic effectors (HSTs) are secreted by *Parastagonospora nodorum*, the causal agent of *P. nodorum* leaf blotch in wheat, that target specific host susceptibility loci resulting in host cell death and promoting the necrotrophic lifestyle of the fungus [142,143,144]. The *SnTox1* interacts with *Snn1* in a light-dependent manner, inducing programmed cell death and enhancing pathogen colonization [145,146]. Notably, *SnTox1* also contains a chitin-binding domain that protects fungal cell walls from plant chitinases and suppresses PRR-mediated immune activation, whereas *Snn1* encodes a wall-associated kinase involved in resistance to biotrophic pathogens, demonstrating how necrotrophic pathogens hijack these pathways for susceptibility [147,148]. Another effector, *SnToxA*, is nearly identical to *PtrToxA* from *Pyrenophora tritici-repentis* and utilizes the same *S*-gene, *Tsn1*, suggesting horizontal gene transfer between pathogens [149]. Additional effectors (*SnTox2*–*SnTox8*) and their corresponding loci (*Snn2*–*Snn8*) have also been identified, several of which trigger light-dependent programmed cell death, further subverting host defenses [149,150,151,152,153,154]. Transcription activator-like (TAL) effectors from *Xanthomonas*, delivered via the type III secretion system, bind specific DNA sequences in host promoters, activating genes that facilitate infection. For instance, TAL effectors such as *AvrBs3* and *PthXo1* induce expression of the sugar transporter gene *OsSWEET14* in rice, promoting bacterial growth by increasing nutrient availability. The *TaNCED* gene in wheat is activated by TAL effector of *Xanthomonas translucens* and results in elevated ABA production and alteration of plant stress responses and water flow, creating conditions that favor pathogen survival and spread [155,156]. Similarly, the victorin toxin from the fungus *Cochliobolus* activates the Arabidopsis gene *LOV1*, converting it from a resistance gene to a susceptibility factor under certain conditions [157]. Additionally, *Pseudomonas* effector HopZ2 targets the Arabidopsis gene *MLO2*, a homolog of the *Mlo* gene in barley, highlighting the role of effector-targeted class 3 *S*-genes in pathogen virulence [158]. Understanding how pathogens exploit *S*-genes is essential for designing effective strategies to improve plant resistance against pathogens.

## 6. Introgression of *S*-Gene for Durable Resistance

### Classical Breeding Approaches

In many crops, over the years classical mutation breeding has been used for the inactivation of *S*-genes [59]. In classical breeding, introgression of these mutated *S*-genes requires multiple backcrosses with the elite parent followed by selfing and selection for resistance and desirable agronomic traits. Such approaches are time consuming, labor-intensive, and even inefficient in eliminating some undesirable traits due to linkage drag. Resistance in plants is commonly assessed through symptom observation in addition to molecular and biochemical analysis, as demonstrated in the case of pea resistance to pea seed-borne mosaic virus (PSbMV) [158]. Traditional breeding for *S*-genes offers the advantage of broad public acceptance. The most well-studied form of durable recessive resistance (*S*-gene mutation) is *eIF4E*-based resistance against potyviruses. The pepper *pvr1/2* gene was the first identified source of recessive resistance to potato virus Y (PVY) and has been successfully utilized for many years. [159,160]. Numerous studies have reported the breakdown of *eIF4E*-based resistance against potyviruses due to frequent mutations in viral proteins (VPs) [161,162,163,164]. Virus replication generally relies on the physical interaction between its VPg and plant *eIF4E* factors [31,165]. Natural variants of *eIF4* factors have been identified in several plants, including lettuce (*Lactuca sativa*), barley (*Hordeum vulgare*), pea (*Pisum sativum*), and pepper (*Capsicum annuum*) and have been successfully introduced into various genotypes [166,167,168,169].

## 7. Targeting *S*-Genes for Durable Resistance

### 7.1. Targeting eIF4 Genes

Potyviruses can overcome inactivated *S*-gene resistance by acquiring new specificity for an alternative *eIF4* isoform or by completely bypassing the need for *eIF4* binding [156]. Only a handful of instances have reported VPs other than VPg being responsible for overcoming the requirement for *eIF4E* [170,171]. Thus, based on the crop and the specific *eIF4E* gene used, resistance to certain potyviruses can be achieved. Chandrasekaran et al. (2016) used CRISPR/Cas9 technology to KO*eIF4E* in cucumber. The edited lines showed resistance to Ipomovirus (CVYV) and potyviruses (ZYMV and PRSV-W) [172]. In another study, *eIF(iso)4E* in *A. thaliana* was targeted using CRISPR/Cas9, resulting in transgene-free plants that showed resistance to turnip mosaic virus (TuMV) [173]. Similarly, the *eIF4G* gene in the susceptible rice variety *Oryza sativa* cv. IR64 was mutated using CRISPR to create new alleles, leading to the development of a resistant variety against rice tungro spherical virus (RTSV) [74,174]. Editing the *eIF4E* gene in wheat, particularly in *TaeiF4E*-*aabbdd* mutants, provided resistance to wheat yellow mosaic virus (WYMV) without affecting growth, resulting in taller plants, delayed heading, and no yield reduction [175]. Base editing of *eIF4E1* by CRISPR/Cas9-cytidine deaminase conferred resistance to clover yellow vein virus (ClYVV) in Arabidopsis [176]. In tomato (cv. M82), *eIF4E* KO conferred resistance to PVYN, but it remained susceptible to PVYO [177]. Similarly, *eIF4E1* editing in the tomato cv. Micro-Tom confers resistance to pepper mottle virus (PepMoV) but not to tobacco etch virus (TEV) [178]. These two above-mentioned studies in tomato demonstrated that CRISPR-mediated inactivation of *eIF4E1* resulted in a narrow resistance spectrum against potyviruses [177,178]. In addition to *eIF4E1*, *eIF4E2* mutants were developed in cherry tomato, showing complete resistance to one PVMV isolate, partial resistance to another, and full susceptibility to both tested Potato virus Y (PVY) isolates [179]. Editing cassava *nCBP1* and *nCBP2* (alternative *eIFE*-like proteins) was shown to reduce the severity and incidence of cassava brown streak (CBS) disease symptoms [180]. CRISPR/Cas9 was employed to mutate the *eIF4E* in the elite tetraploid potato cultivar ‘Kruda’, resulting in enhanced resistance to PVY. The edited lines exhibited broad-spectrum resistance [181]. Resistance has been developed in melon against Moroccan watermelon mosaic virus (MWMV) by targeting the gene encoding the cap-binding protein eIF4E [182,183]. CRISPR-mediated mutations created in *eIF4E* and cucumber plants resistant to watermelon mosaic virus (WMV), papaya ringspot virus (PRSV), and zucchini yellow mosaic virus (ZYMV) have been developed [184]. In another study, KOof *CleIF4E1* in watermelon provided resistance to ZYMV, however, the mutants showed developmental defects, including altered plant growth, abnormal leaf morphology, and reduced yield [185].

### 7.2. Targeting Mlo Genes

The *Mlo* gene family encodes membrane-associated proteins that act as negative regulators of plant immunity, making them ideal targets for *S*-gene editing. Loss-of-function mutations in *Mlo* genes confer durable and broad-spectrum resistance to PM, a widespread fungal disease that severely affects many crops [11,186]. Although natural resistance to PM is typically polygenic, modern gene-editing tools like CRISPR/Cas9 enable the precise editing of *Mlo* genes, offering a targeted approach for developing resistant cultivars. Tek et al. used CRISPR/Cas9 to investigate the function of genes responsible for *mlo* resistance to PM [187]. CRISPR/Cas9 GE of the *Mlo* gene has been successfully implemented in wheat, barley, grapevine, cucumber, tomato, and soybean [22,188,189,190,191,192,193]. In 2014, Wang et al. used the CRISPR/Cas9 system to target the *TaMLOA1* allele in the wheat cultivar Kenong 199. The mutants exhibited robust and broad-spectrum resistance to PM along with undesirable pleiotropic effects [22]. Similarly, Nekrasov et al. targeted the *SlMlo1* loci and developed a variety named “Tomelo” (genome-edited tomato), which conferred resistance to the PM pathogen *Oidium neolycopersici* [191]. In addition, two susceptibility genes *SlPelo* and *SlMlo1* have been targeted in tomato (BN-86 cultivar) using multiple sgRNAs and KOsgenerated for *SlMlo1* showed complete resistance to PM [192]. In 2022, a wheat variety, Tamlo-R32, having targeted deletion (304 Kb) in the *MLO-B1* locus, has been developed using CRISPR, which confers strong resistance to PM without compromising plant growth or yield [188]. In grapevine, four *VvMLO3*-edited lines generated using CRISPR/Cas9 exhibited enhanced resistance to PM along with cell wall appositions in edited lines compared to WT lines [189]. In another study, CRISPR/Cas9 was used to develop PM-resistant cucumber by targeting *CsaMLO1*, *CsaMLO8*, and *CsaMLO11* genes. Mutation in *CsaMLO8* and *CsaMLO1/CsaMLO11* corresponded to pre-invasion and post-invasion defense responses (HRs), respectively [187]. Recently, Shnaider et al. (2023) targeted *CsaMLO8* using CRISPR technology to develop PM resistance in the susceptible cucumber cultivar Ilan [190]. In another study, researchers used a dual gRNA CRISPR construct to simultaneously KO four *MLO* homologs (*GmMLO02*, *GmMLO19*, *GmMLO20*, and *GmMLO23*) in the Vietnamese soybean elite cultivar DT26, which conferred increased resistance to PM [193].

### 7.3. Targeting SWEET Family Genes

Targeting *SWEET* genes has proven effective in controlling bacterial infections in crops, particularly bacterial blight in rice [194]. Several studies have reported CRISPR/Cas9-mediated editing of the *OsSWEET* gene promoters to enhance resistance against *Xanthomonas oryzae pv. oryzae* (*Xoo*), the causative agent of bacterial blight [195,196]. In a notable study, Zafar et al. (2020) edited the effector binding elements (EBEs) in the *OsSWEET14* promoter that are recognized by three TALEs (AvrXa7, PthXo3, and TalF). This promoter editing was performed in four elite super basmati rice lines, resulting in enhanced resistance to *Xoo* [197]. In another study, CRISPR mediated KOof *OsSWEET14* in rice cultivar Zhonghua 11 conferred strong resistance to both African and Asian *Xoo* strains [198]. CRISPR/Cas9-mediated editing of the *OsSWEET11* promoter in Mentik Wangi rice (a local variety of central Java) conferred bacterial blight resistance without altering key agronomic traits [199]. Recently, cytidine and adenine base editors based on CRISPR/Cas9 were employed to modify the TalC effector-binding element in the *OsSWEET14* promoter of rice cultivars IR24, Kitaake, and Zhonghua 11. C-to-T edits disrupted *OsSWEET14* induction, conferring resistance to African *Xoo* strains, while A-to-G edits maintained susceptibility without any off-targets effects [200].

### 7.4. Targeting Other S-Genes

In tomatoes, *StDMR6-1* ortholog has been mutated using CRISPR/Cas9 and mutated lines showed increased resistance to *P. capsici* and *P. syringae* pv. tomato, indicating the broad-spectrum disease resistance role of *DMR6-1* [201]. CRISPR/Cas9-mediated multiplex GE of the *BnWRKY70* gene in *B. napus* resulted in mutants with enhanced resistance to *Sclerotinia sclerotiorum* [33]. In Arabidopsis, mutants of enhanced disease resistance 1 (*EDR1*) gene provide resistance to *Erysiphe cichoracearum*, a causal agent of PM [202]. Therefore, *EDR1* serves as a promising target *S*-gene for enhancing resistance to PM. Using CRISPR/Cas9 to simultaneously edit all homologs of wheat, *TaEDR1* produced mutant plants resistant to *Blumeria graminis* f. sp. tritici (Bgt). Interestingly, these edited plants showed no adverse effects on growth or development [203]. In apple, the *MdDIPM4* gene, a member of the DIPM family associated with susceptibility to fire blight, was targeted using CRISPR technology, resulting in edited cultivars with enhanced resistance to the disease [204]. In addition to *MLO*, powdery mildew resistance 4 (*AtPMR4*) has been identified as another *S*-gene in Arabidopsis. Recently, Martinez et al. used CRISPR/Cas9 to edit the *SlPMR4* gene (*AtPMR4* ortholog) in tomato. The edited T2 mutant plants exhibited partial resistance to *Oidium neolycopersici* with growth penalties [205]. In potato, CRISPR/Cas9 was used to generate functional KOs of *StDND1*, *StCHL1*, and *StDMR6-1*, resulting in increased resistance to late blight. Among these, *StDMR6-1* and *StCHL1* mutants showed no adverse growth effects, making them promising candidates for disease-resistant crop development [206]. In addition, simultaneous silencing of miR482b and miR482c provided enhanced resistance to *P. infestans* [207]. In sweet basil, Zhang et al. (2021) used CRISPR/Cas9 to edit the orthologous *ObHSK* gene, generating transgene-free plants. The edited lines exhibited normal growth under greenhouse conditions and showed enhanced resistance to *Peronospora belbahrii* compared to WT [208]. Cha et al. recently targeted the nucleoredoxin (*SlNRX1*) gene in tomato through CRISPR/Cas9 technology, and the mutants displayed enhanced resistance against the fungal pathogen *Alternaria brassicicola*. [209]. Similarly, multiplex CRISPR/Cas9 editing of *miR482b* and *miR482c* (acting as susceptibility factors) in tomato exhibited reduced disease symptoms and increased yield [210,211,212]. A new susceptibility gene, *StPM1*, was identified in *Solanum tuberosum* by Bi et al. (2023), and CRISPR/Cas9-mediated KOof *StPM1* conferred resistance to *Phytophthora* without impacting plant growth or development [213]. CRISPR/Cas9-mediated KOof the rice *S*-gene *OsHPP04* enhanced resistance to *Meloidogyne graminicola* without affecting plant growth. The transgene-free mutants showed stronger immune responses, showing the feasibility of *S*-gene editing for developing nematode-resistant crops [214]. A summary of successful editing events targeting *S*-genes in various crops is provided in Table 2.

## 8. Global Policies and Regulatory Approaches for GE Development and Commercialization

GE stands out as the breakthrough technology of the decade in agriculture for crop improvement. Unlike traditional genetically modified organisms (GMOs), genome-edited crops may not contain foreign DNA, blurring the lines for regulatory frameworks. As technology use expands, so does the need for robust governance to ensure biosafety, transparency, and equitable benefit sharing. In many countries, GMOs and gene-edited crops are often governed by the same sets of rules and regulations, despite their fundamental differences [239]. Globally, regulatory frameworks for GMOs generally follow two primary approaches, product-based regulations (focus is on the safety and characteristics of the final product, regardless of how it was developed) and process-based regulations (focusing on the techniques used to create GMOs, especially when introducing new traits). Differences in these regulations partly reflect varying national priorities and the fact that some countries have not signed the Cartagena Protocol on Biosafety (CPB). For instance, the EU’s definition of GMOs as “not naturally altered” impacted the public perception as a whole towards GE technologies [240]. In 2018, the European Court of Justice ruled that genome-edited organisms are subject to the GMO Directive, Callaway, 2018 (Confédération paysanne and others v. Premier ministre and Ministre de l’Agriculture de l’Agroalimentaire et de la Forêt, 2018) [241]. The European Union follows a process-based approach, classifying most GE crops as GMOs. This results in extensive labeling and traceability requirements, effectively silently negating rapid adoption, despite ongoing debates about revising the framework for new genomic techniques (NGTs). Likewise, Malaysia and several African nations have strict GMO style regulations, resulting in delayed approvals for GE crops [242].

In contrast to the EU, the U.S. has adopted a product-based regulatory approach. The USDA does not subject certain genome-edited crops (SDN 1, SDN 2) to the same scrutiny as GMOs, provided they do not contain foreign DNA and could have been developed through conventional breeding [242]. In December 2024, a federal judge overturned the SECURE Rule, restoring pre-2020 regulations. While previously approved crops remain unaffected, the ruling adds complexity and cost to new products, posing challenges for smaller developers. A CRISPR/Cas9-edited white button mushroom resistant to browning was the first genome-edited crop to be exempt from USDA regulations [243]. The USDA has consistently supported research on CRISPR/Cas9 technology across various crops [244,245]. The US adopts a product-based approach, where crops with minor edits (SDN 1, SDN 2) are exempt from GMO regulations, enabling faster approvals and lower costs.

**Figure 5:**
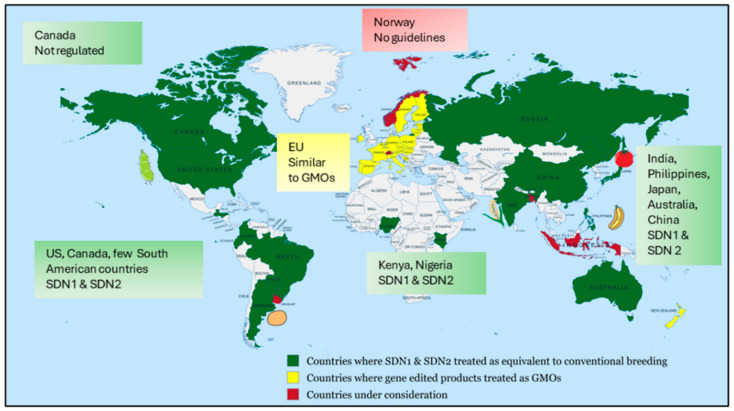
Global regulatory landscape for genome-edited crops.

Similar regulation to the USA has been implemented by Canada and four South American countries that classified genome-edited crops (SDN-1, -2) as equivalent to conventional breeds [242]. Similarly, India’s regulatory framework for genome-edited crops was updated in 2022, exempting plants without foreign gene insertions from being classified as transgenic provided they undergo a safety assessment (Ministry of Environment, Forest and Climate Change MoEFCC, Govt. of India, 2022). India exempts genome-edited plants developed through SDN 1 and SDN 2 techniques (Guidelines for the Safety Assessment of Genome Edited Plants, 2022). In May 2025, India approved its first genome-edited rice varieties, DRR Dhan 100 (Kamala) and Pusa DST Rice 1. In Australia, the 2019 amendments specify that organisms modified using SDN 1 technique are generally exempt from regulation under the Act, as such changes are considered comparable to those occurring naturally or through conventional breeding. The African Union’s Agenda 2063 emphasizes the use of GE to enhance agricultural productivity and crop resilience. So far, two countries (Nigeria and Kenya) have implemented regulations for a case-by-case review of genome-edited crops [246] (Figure 5).

Countries like Argentina, Brazil, and Japan, have adopted more flexible regulatory frameworks that distinguish GE crops from traditional GMOs where no foreign genes are introduced [234,235]. In 2022, the Ministry of Agriculture and Rural Affairs in China released safety guidelines for the evaluation of genome-edited plants for agriculture. Recently, in May 2024, the Chinese government approved gene-edited wheat for the first time, marking a significant step toward the adoption of genome-editing food crops. In summer 2024, the Philippines’ Department of Agriculture and Bureau of Plant Industry classified genome-edited bananas as non-GMO, approving them for import and cultivation (www.isaaa.org). However, such varying national approaches pose challenges for international trade and innovation. Generally, crops produced by CRISPR/Cas9 and other gene editing methods are challenging traditional definitions of GMOs and pushing regulatory bodies to reconsider existing frameworks. Regulatory bodies worldwide are still adjusting to the fast-paced advancements in this technology. Therefore, despite existing legal hurdles, researchers, investors, and consumers should continue to support and prioritize the development and study of improved and beneficial crops. NBTs have the potential to create more resilient, nutritious, and high-yielding plants that can meet the growing global food demand and address challenges like climate change and disease pressures.

## 9. Challenges and Future Perspectives

While targeting *S*-genes via GE has proven to be a promising strategy for enhancing disease resistance, there are several challenges that hinder its universal application. First, many *S*-genes play dual roles in plant physiology, and their inactivation may lead to unintended pleiotropic effects. For example, the *eIF4E* gene, essential for potyvirus replication, is also involved in basic cellular processes, and its modification can result in growth penalties. Another challenge is the potential for resistance breakdown due to the adaptive evolution of pathogens. For instance, potyviruses have been shown to evolve new VPg variants capable of overcoming *eIF4E*-based resistance. Moreover, while *S*-gene resistance tends to be durable and broad-spectrum, its recessive nature complicates its integration into elite lines through conventional breeding. Another major challenge resides in the widespread public opinion that fails to distinguish between genetically modified organisms (GMOs) and genome-edited crops, often viewing both as equally harmful to the environment and human health. This misconception is compounded by the lack of a unified global regulatory framework that clearly differentiates genome-edited crops from GMOs and facilitates their production and commercialization [247]. Regulatory frameworks also remain inconsistent globally, for example, the European Union and New Zealand currently regulates gene-edited crops under strict GMO legislation, in contrast to product-based approaches in North and South America (such as the USA, Colombia, Canada, and Argentina), along with several in Asia (including India, China, and Japan), and Australia [248]. This disparity creates obstacles for global commercialization and international trade.

Overall, the manipulation of plant *S*-genes presents a promising avenue for enhancing disease resistance in crops, but it also carries the risk of negatively impacting vital plant processes due to the native functions these genes often perform. Many *S*-genes are integral to essential physiological functions like sugar allocation, mediated by *SWEET* transporters, or the regulation of defense signaling by various kinases. Therefore, altering these genes can lead to undesirable trade-offs, including reduced yield, impaired growth, or decreased tolerance to environmental stresses. To circumvent trade-offs it is important to understand the roles of *S*-genes in physiology and development [59]. Looking ahead, several emerging innovations offer a pathway to overcome these hurdles and further harness the potential of *S*-gene editing. Recent advancements in GE technologies such as base editing and prime editing provide powerful tools to precisely modify *S*-genes, minimizing unintended negative consequences on plant fitness [59,249]. These technologies allow researchers to make targeted genetic alterations that can confer disease resistance while preserving the essential native functions of the genes. Strategies such as cis-regulatory editing, modifying promoter regions instead of coding sequences, offer a way to attenuate gene expression rather than abolish function entirely [250]. The integration of multi-omics approaches, such as transcriptomics, proteomics, and metabolomics, with machine learning techniques is significantly accelerating the identification and prioritization of candidate *S*-genes. These advanced computational methods can analyze vast datasets to uncover complex gene regulatory networks and predict the functional impact of *S*-gene modifications. This allows researchers to more accurately identify *S*-genes whose perturbation would lead to beneficial resistance traits while minimizing undesirable trade-offs. By leveraging these integrated approaches, plant breeders can make more informed decisions, leading to the development of more resilient and sustainable crops.

Additionally, multiplex CRISPR platforms are enabling the simultaneous editing of several susceptibility loci, which can significantly enhance resistance durability. Finally, harmonization of international regulatory policies and increased public engagement are essential to reduce barriers to market entry and foster societal trust in genome-edited crops. Lastly, the combination of *S*-gene editing with sustainable agricultural practices, can provide a holistic strategy to achieve resilient, productive, and environmentally friendly food systems in the face of climate change and increasing disease pressures.

## Figures and Tables

**Table 1:** Databases and computational tools for susceptibility (*S*) gene identification and functional prediction (all links accessed on 27 September 2025).

Database /Software	Domain Name	Uses	Framework & Model Type	References
PRGdb 4.0	http://prgdb.org/prgdb4/	To predict resistance genes and investigate gene expression under specific plant–pathogen conditions	-	García et al., 2022 [84]
webTWAS	http://www.webtwas.net/#/	A database of potential disease *S*-genes identified through transcriptome-wide association studies.	-	Ca et al., 2022 [85]
HMMER	www.hmmer.org	General profile-HMM database search and alignment tool	-	Finn et al., 2011 [86]
PSIBLAST	https://www.ebi.ac.uk/jdispatcher/sss/psiblast	Progressive BLAST algorithm employing PSSMs	-	Altschul et al., 1997 [87]
Hhblits	http://www.github.com/soedinglab/hh-suite	Identification of distant sequence homologs	-	Remmert et al., 2012 [88]
SignalP	http://www.cbs.dtu.dk/services/SignalP	Prediction of secreted proteins by detecting N-terminal signal peptides, a common initial step for effector prediction	-	Teufel et al., 2022 [89]
TargetP	http://www.cbs.dtu.dk/services/TargetP	General prediction of protein subcellular or extracellular localization	-	Emanuelsson et al., 2007 [90]
TMHMM	http://www.cbs.dtu.dk/services/TMHMM	Prediction of transmembrane domains in proteins	-	Chen 2003 [91]
LOCALIZER	https://localizer.csiro.au	Prediction of localization within host plant cells	-	Sperschneider et al., 2017 [92]
ApoplastP	https://apoplastp.csiro.au	Prediction of proteins localized in the plant apoplast	-	Sperschneider et al., 2018 [93]
dbSWEET	https://ngdc.cncb.ac.cn/databasecommons/database/id/6133	SWEET transporter prediction	Python/HTML	Gupta et al., 2018 [94]
EffectorP	https://effectorp.csiro.au/	Predict fungal and oomycete effectors and classify localization	Naïve Bayes + C4.5 Decision Trees (Ensemble)	Sperschneider et al., 2021 [95]
DeepGO	https://deepgo.cbrc.kaust.edu.sa/deepgo/	Predicts protein function to prioritize *S*-gene candidates	Convolutional Neural Networks (CNNs) and Deep Neural Networks (DNN)	Maxat et al., 2018 [96]
GeneMANIA	http://genemania.org	Predict gene function using network topology, co-expression, and orthologous features	Label propagation and adaptive network weighting	Warde-Farley et al., 2010 [97]
BLAST	https://blast.ncbi.nlm.nih.gov/Blast.cgi	*S*-gene (homologous) identification	Alignment-based	Altschul et al., 1990 [98]
InterProScan	https://www.ebi.ac.uk/interpro/search/sequence/	Identification of *S*-genes based on protein domains or families	Rule-based domain annotation	Blum et al., 2025 [99]
Effhunter	https://github.com/GisCarreon/EffHunter_v.1.0	Fungal effector prediction	Rule-driven pipeline	Carreón-Anguiano et al., 2020 [100]
Pathoplant	https://ngdc.cncb.ac.cn/databasecommons/database/id/1500	Reference for Plant-pathogen interaction	Relational Database	Bülow et al., 2004 [101]
InterSPPI-AraPathogen2.0	http://zzdlab.com/intersppi/arapathogen/	Predict *Arabidopsis*-pathogen interaction	XGBoost (Extreme Gradient Boosting)	Lei et al., 2023 [102]
HPIDB3.0	https://cales.arizona.edu/hpidb/	Host–pathogen protein Interaction	Relational Database	Ammari et al., 2016 [103]
PHIbase	http://www.phi-base.org/	Host–pathogen interaction database	Curated database	Urban et al. 2022 [104]
Phytozome	https://phytozome-next.jgi.doe.gov	Predict gene families and evolutionary relationships	Distance- and rule-based comparative genomics database	Goodstein et al., 2012 [105]
Plant Ensembl	https://plants.ensembl.org/	Gene sequences and orthologs	Rest & Perl API	Bolser et al., 2017 [106]
TAIR (The Arabidopsis Information Resource)	https://www.arabidopsis.org	Reference for model species to identify orthologs	Curated resources	Reiser et al., 2024 [107]
Plant Resistance Gene Database (PRGdb)	http://www.prgdb.org/prgdb4/	Predicts primarily *R*-genes, but useful for cross-referencing *S*-gene candidates	Domain scoring and rule-based thresholds	García et al., 2021 [84]
OrthoMCL	https://orthomcl.org	Gene family clustering and ortholog identification	Markov Cluster Algorithm (MCL)	Li et al., 2003 [108]
Gene Expression Omnibus (GEO)	https://www.ncbi.nlm.nih.gov/geo/	Identify differentially expressed genes as *S*-gene candidates	Expression repository	Barrett et al., 2013 [109]

**Table 2:** List of *S*-gene editing events in various crops.

Crop	*S*-Gene	Disease	Associated Pathogen	Function of *S*-Gene	Result/Outcome	Reference
*Arabidopsis thaliana*	*elF(iso)4E*	Viral disease	Yellow mosaic virus (YMV)	Recessive resistance alleles against potyviruses	Induced mutation in *elF(iso)4E* imparts complete resistance	Pyott et al., 2016 [173]
Banana (*Musa acuminate*)	*MusaDMR6*	Xanthomonas wilt	*Xanthomonas campestris* pv. *musacearum*	Salicylic acid degradation (SA-5-hydroxylase), suppresses host immunity	Improved resistance with no detrimental impact on plant	Tripathi et al., 2021 [215]
Cassava (*Manihot esculenta*)	*nCBP1* and *nCBP2*	Viral resistance	Cassava brown streak virus (CBSV)	Susceptibility factor	Resilience to viral disease	Gomez et al., 2019 [180]
Cucumber (*Cucumis sativus L*.)	*CsaMLO1*, *CsaMLO8*, *CsaMLO11*	Powdery mildew (PM)	*Podosphaera xanthii*	Negative regulator of pre- (*CsaMLO8*) and post-invasive (*CsaMLO1*, *CsaMLO11*) defense	*CsaMLO8* loss-of-function conferred highest penetration resistance; with *CsaMLO1* and *CsaMLO11* double mutations seemed good candidates for HR-based resistance against PM pathogen	Tek et al., 2022 [187], Ma et al., 2024 [216]
	*elF4E*	Viral diseases	Potyviruses	Encodes a translation initiation factor that interacts with potyviral VPg proteins for viral infection.	Loss-of-function mutations disrupt viral replication, conferring resistance. Homozygous elF4E_1DEL and elF4E_1-3DEL mutants showed complete resistance to watermelon mosaic virus (WMV), papaya ringspot virus (PRSV), and zucchini yellow mosaic virus (ZYMV)	Chandrasekaran et al., 2016 [172] Fidan et al., 2023 [184]
Grapevine (*Vitis vinifera*)	*VvDMR6-1* and *VvDMR6-2*	Downy mildew (DM)	*Plasmopara viticola*	Negative regulator of plant immunity	Reduced severity, simultaneous editing of both genes is required for reduced susceptibility	Giacomelli et al., 2023 [217] Djennane et al., 2023 [218]
Melon (*Cucumis melo*)	*elF4E*	Viral disease	Ringspot mosaic virus-W (RMV-W)	Encodes a cap-binding protein essential for translation initiation. It acts as a proviral factor by facilitating viral RNA translation and in sexual development	Loss-of-function mutations confer virus resistance but also cause male sterility	Shirazi et al., 2023 [182] Pechar et al., 2022 [183]
	*Prv*	Viral disease	PRSV-W	NLR resistance gene required for PRSV immunity	Knockout mutants lost PRSV resistance; one allele (*prvΔ154*) exhibited an autoimmune dwarf phenotype suppressed at high temperatures	Nizan et al., 2023 [219]
Potato (*Solanum tuberosum*)	*StNRL1*	Late blight	*Phytophthora infestans*	Forms a protein complex with the *P. infestans* effector, leading to degradation of SWAP70, a positive regulator of cell death	Knockdown enhanced resistance to *P. infestans* but increased susceptibility to *Alternaria alternata*, suggesting a dual role in defense	Norouzi et al., 2024 [220]
	*eIF4E*	Virus resistance	Potato virus Y (PVY)	Translation initiation factor required by PVY via VPg interaction	Knockout of *eIF4E* conferred broad-spectrum resistance to PVY with significantly delayed/reduced virus titer, and no adverse growth or developmental abnormalities	Noureen et al., 2022 [181]
	*StPM1*	Late blight	*Phytophthora infestans*	Encodes a plasma membrane protein that interacts with StRbohC, promoting its degradation and negatively regulating reactive oxygen species (ROS) production	Knockout mutants exhibited enhanced resistance to *P. infestans* without growth penalties; increased expression of defense-related genes.	Bi et al., 2024 [213]
Rapeseed (*Brassica napus*)	*BnHva22c*	Stem striping	*Verticillium longisporum*	Susceptibility factor	Improved resistance	Ye et al., 2024 [221]
	*BnaA05.RLK902*	Stem rot disease (SRD), grey mold disease (GMD)	*Sclerotinia sclerotiorum*, *Botrytis cinera*	Plasma membrane RLK negatively regulating necrotrophic immunity	Resistance to both diseases; no growth trade-off	Zhao et al., 2024 [222]
	*BnWRKY70*	Sclerotinia stem rot	*Sclerotinia sclerotiorum*	Transcription factor; negatively regulates defense response against *S. sclerotiorum*	Knockouts showed enhanced resistance; overexpression increased susceptibility	Sun et al., 2018 [33]
Sweet basil (*Ocimum basilicum*)	*ObDMR6*	DM	*Hyaloperonospora arabidopsidis*	SA-5-hydroxylase homolog that degrades salicylic acid, lowering host immunity and promoting pathogen growth	Knockout of *ObDMR6* led to enhanced resistance with reduced sporangia and pathogen biomass	Hasley et al., 2021 [223]
Tomato (*Solanum lycopersicum*)	*SlMlo1*	PM	*Oidium neolycopersici*	Susceptibility factor for fungal PM	Knockout of *SlMlo1* conferred complete resistance to PM	Pramanik et al., 2021 [192]
	*SlPMR4*	PM	*Oidium neolycopersici (On)*	Encodes callose synthase at fungal penetration site, which is exploited by pathogen	Disrupting *SlPMR4* reduces susceptibility by increasing HR	Santillán Martínez et al., 2020 [205]
	*SlJAZ2*	Bacterial speck	*Pseudomonas syringae* pv. Tomato (*Pto*) DC300	Encodes coronatine co-receptor in stomatal guard cell that facilitates pathogen colonization	Knockout resulted in increased resistance	Ortigosa et al., 2019 [224]
	*SlNRX1*	Bacterial speck	*Alternaria brassicicola* and *Pseudomonas syringae* pv. maculicola	Mutation boosting the salicylic pathway for immunity	Enhances plant immunity by negatively modulating the expression of the gene	Cha et al., 2023 [209]
	*miR482b*, *miR482c*	Late blight	*Phytophthora infestans*	Repress NBS-LRR defense gene transcripts	CRISPR knockout of miR482b + miR482c conferred resistance to *P. infestans.* Double mutants showed stronger resistance than miR482b alone	Hong et al., 2021 [225]
	*eIF4E1*	Virus resistance	Pepper mottle virus (PepMoV)	Translation initiation factor (eIF4E1) is hijacked by potyviruses via VPg interaction to facilitate viral translation	Knockout conferred strong resistance to PepMoV, with no resistance to tobacco etch virus (TEV) and no adverse growth defects	Atarashi et al., 2020 [177] Yoon et al., 2020 [178]
	*SlDMR6-1*	Resistance to bacterial, oomycete, and fungal pathogens	*Pseudomonas syringae*, *Xanthomonas gardneri*, *Xanthomonas perforans*, *Phytophthora capsici*, *Pseudoidium neolycopersici*	Encodes a 2-oxoglutarate Fe (II)-dependent dioxygenase that acts as a salicylic acid (SA) 5-hydroxylase, converting SA to 2,5-dihydroxybenzoic acid	Knockout of the tomato susceptibility gene *SlDMR6-1* significantly enhances resistance against a broad range of pathogens, including bacteria, oomycetes, and fungi	Thomazella et al., 2021 [226]
	*eIF4E2*	Virus resistance	Pepper veinal mottle virus (PVMV)	Loss-of-function prevents PVMV from hijacking the cap-binding complex	Knockout of *eIF4E2* conferred resistance to PVMV	Kuroiwa et al., 2022 [179]
	*TPL1*, *TPL2*	Fusarium wilt	*Fusarium oxysporum*	Encode transcriptional co-repressors that interact with the fungal SIX8 effector, promoting disease susceptibility	TPL1 knockout reduced susceptibility; TPL1/TPL2 double knockout provided higher resistance	Aadlers et al., 2023 [227]
Rice (*Oryza sativa*)	*OsSWEET14*, *Os SWEET11*, *OsSWEET13*	Bacterial leaf blight	*Xanthomonas oryzae* pv. *oryzae* (Xoo)	Encodes a sugar transporter that is used by *Xoo* TALE (transcription-ativator-like effectors) (e.g., AvrXa7, PthXo3/2, TalC, Tal5) for nutrient acquisition, promoting pathogen virulence	Mutations in TALE-binding elements (EBEs) prevent pathogen-induced expression and confer resistance	Blanvillain-Baufume et al., 2016 [228] Zhou et al., 2015 [229] Olivia et al., 2019 [195] Zafer et al., 2020 [197] Aji et al., 2025 [199]; Li et al., 2025 [200]
	*EBETal6b of OsSWEET11a*	Bacterial blight	*Xanthomonas oryzae pv. oryzae*	Sugar transporter	Rapid resistance response that blocked disease development	Xu et al., 2024 [230]
	*RBL1^Δ^12*	Rice blast	*Magnaporthe oryzae*	Associated with effector secretion and fungal infection	Confers broad-spectrum disease resistance	Sha et al., 2023 [231]
	*OsHPP04*	Root-knot nematode	*Meloidogyne graminicola*	Act as a negative regulator of host immunity against rice root-knot nematode (*Meloidogyne graminicola*)	Enhance resistance with no adverse effect on main agronomic traits	Huang et al., 2023 [214] Song et al., 2021 [232]
	*Pi21* and *Bsr-d1*	Rice blast	*Magnaporthe oryzae*	Suppresses basal defense mechanisms	The triple mutant had much higher resistance to both *M. oryzae* and Xoo than the single mutants	Tao et al., 2021 [233]
	*Xa5*	Bacterial blight	*Xanthomonas oryzae* pv. *oryzae*	Susceptibility factor for disease	Triple mutant with higher resistance	Tao et al., 2021 [233]
	*eIF4G*	RTD (Rice tungro disease)	Rice tungro spherical virus (RTSV)	Translation initiation factor exploited by viral RNA for protein synthesis and infection	Resistance to RTSV without growth penalty	Macovei et al., 2018 [174] Cao et al., 2020 [74]
	*OsERF922*	Rice blast	*Magnaporthe oryzae*	Encodes an ERF transcription factor that negatively regulates plant defense	Reduced blast lesion formation without affecting agronomic traits; heritable resistance in T1 and T2 generations	Wang et al., 2016 [234]
Wanjincheng orange (*Citrus sinensis Osbeck*)	*CsLOB1*	Citrus canker	Zucchini yellow mosaic virus (ZYMV)	Encodes a transcription factor activated by Xcc effector PthA4, promoting disease development	Promoter editing of *CsLOB1* enhanced resistance; no symptoms in some mutants	Peng et al., 2017 [235]
Watermelon (*Citrullus lanatus*)	*Clpsk1*	Fusarium wilt	*Fusarium oxysporum* f. sp. *Niveum* (FON)	Encodes the precursor of phytosulfokine (PSK), a peptide hormone that negatively regulates plant immunity	Loss-of-function mutations enhance resistance to *FON*	Zhang et al., 2020 [236]
	*CleIF4E1*	Viral disease	ZYMV, Cucumber green mottled mosaic virus (CGMMV)	Major recessive factors for many viruses (especially potyviruses)	Mutant line exhibited resistance to ZYMV but not CGMMV, with developmental defects and reduced yield	Li et al., 2024 [185]
Wheat (*Triticum aestivum*)	*TaMLO-A1*, *TaMLO-B1*, *TaMLO-D1*	PM	*Blumeria graminis* f. sp. *tritici* (Bgt)	MLO proteins negatively regulate defense	Triple-mutant plants exhibited heritable, broad-spectrum resistance to PM	Li et al., 2022 [188]
	*SlMlo1*	PM	*Blumeria graminis* f. sp. *tritici* (Bgt)	MLO proteins negatively regulate defense	Complete resistance to PM	Nekrasov et al. 2017 [191] Pramanik et al., 2021 [192]
	*TaEDR1* (homoeo alleles in A, B, and D subgenomes)	PM	*Blumeria graminis* f. sp. *tritici* (Bgt)	Negative regulator of PM resistance	Triple-mutant Taedr1 wheat plants showed resistance to powdery mildew with no off-target mutations or pleiotropic effects	Zhang et al., 2017 [203]
	*TaWRKY19*	Stripe rust	*Puccinia striiformis* f. sp. *tritici* (Pst)	WRKY transcription factor; negative regulator of plant immune response	Knockout resulted in strong resistance to stripe rust	Wang et al., 2022 [237]
	*TaGW2*	Leaf rust	*Puccinia triticina* Eriksson *(Pt)*	E3 ubiquitin ligase; negative regulator of wheat grain width and weight	Knockout led to resistance to leaf rust with increased grain width and weight	Liu et al., 2024 [238]
	*TaPslPK1*	Stripe rust	*Puccinia striiformis* f. sp. *tritici* (Pst)	Encodes a receptor-like cytoplasmic kinase targeted by the fungal effector PsSpg1, promoting virulence by phosphorylating TaCBF1d and modulating gene expression	Knockout of *TaPsIPK1* conferred broad-spectrum resistance against Pst without affecting agronomic traits in field tests	Wang et al., 2022 [237]

## Data Availability

No new data were generated or analyzed in this study. All data referenced are publicly available in the cited literature.
